# The Florida Medical and Surgical Journal

**Published:** 1886-02

**Authors:** 


THE FLORIDA MEDICAL AND SURGICAL JOURNAL, From the land of fruits and flower comes as bright a blossom, added to the boquet of medical journalism, as has been contributed by any State; a blossom which is not only fragrant with that essence of intellectuality which men call genius, (*hereditary* in this instance, and scintillating in the writing of a worthy son of a noble sire,—a sire said to have been born with a pen in hand), but redolent of promise of much and good fruit. We welcome it with all the warmth of our southern nature, and will watch its unfolding and fruition with a pleased and interested eye. From this land of *Summers* perennial, the world will expect "spicy" breezes,—and, by the way our friends are working, and, by the way they have begun, we predict there will be none disappointed. We beg to acknowledge as graceful a compliment at the hands of our Florida fledgeling as it has ever been our good fortune to receive, and here and now, with the assurance that its sincerity has sunk it deep into our heart, we make our prettiest bow, and elevate our new silk hat, which was presented to us Christmas day. We are yours to command, Brother Summers.

